# Quantitative analysis of bony and soft tissue contributions to varus deformity across knee osteoarthritis stages

**DOI:** 10.1002/jeo2.70704

**Published:** 2026-04-06

**Authors:** Kyota Ishibashi, Ryoto Kura, Ryo Tomita, Eiji Sasaki, Hikaru Kristi Ishibashi, Yuka Kimura, Eiichi Tsuda, Yasuyuki Ishibashi

**Affiliations:** ^1^ Department of Orthopaedic Surgery Hirosaki University Graduate School of Medicine Hirosaki Japan; ^2^ Department of Rehabilitation Medicine Hirosaki University Graduate School of Medicine Hirosaki Japan

**Keywords:** hip–knee–ankle angle, joint line convergence angle, knee osteoarthritis, lower limb alignment

## Abstract

**Purpose:**

To investigate the relationships among lower limb alignment parameters and quantitatively clarify the stage‐dependent contributions of bony morphology and soft tissue laxity to varus deformity across knee osteoarthritis stages.

**Methods:**

We analysed 1346 knees of 673 participants in a community‐based cohort. Radiographic parameters, including the hip–knee–ankle angle (HKAA), weight‐bearing line ratio (WBLR), femorotibial angle (FTA), medial proximal tibial angle (MPTA), lateral distal femoral angle (LDFA) and joint line convergence angle, were measured using full‐length standing radiographs. Spearman's correlation coefficients and linear regression analyses were performed for the overall cohort and stratified subgroups: early‐ (Kellgren–Lawrence Grade [KLG] 0–1), progressive‐ (KLG 2) and end‐stage (KLG 3–4).

**Results:**

In the overall cohort, the HKAA exhibited robust correlations with WBLR (*r* = −0.99, *p* < 0.001) and FTA (*r* = 0.86, *p* < 0.001), validating FTA as a reliable global alignment surrogate. Stage‐dependent analysis revealed a change in the relative associations among determinants of deformity. In the early‐ and progressive‐stage groups, varus alignment was predominantly associated with bony parameters (MPTA and LDFA). Conversely, in the end‐stage group, the correlation between the joint line convergence angle and varus deformity strengthened significantly (*r* = 0.61 vs. *r* = 0.20 in the early stage).

**Conclusions:**

While bony deformity is a consistent factor, the contribution of soft tissue laxity (joint line convergence angle) becomes substantial in end‐stage osteoarthritis. In end‐stage osteoarthritis, the joint line convergence angle is a key alignment determinant. Surgical planning for joint‐preserving procedures, including high tibial osteotomy, must account for this quantitative confirmation of stage‐dependent differences to prevent reducible soft tissue component overcorrection.

**Level of Evidence:**

Level III.

**Abbreviations**
2D: two‐dimensional
AP: anteroposterior
BMI: body mass index
FTA: femorotibial angle
HKAA: hip–knee–ankle angle
HTO: high tibial osteotomy
ICC: intraclass correlation coefficient
JLCA: joint line convergence angle
KLG: Kellgren–Lawrence Grade
LDFA: lateral distal femoral angle
MPTA: medial proximal tibial angle
OA: osteoarthritis
WBLR: weight‐bearing line ratio

## INTRODUCTION

Varus alignment is widely recognized as a primary risk factor for medial compartment knee osteoarthritis (OA) progression and is a critical parameter in surgical decision‐making for high tibial osteotomy (HTO) [3, 15, 17, 25]. Accurate lower limb alignment assessment, typically quantified by the hip–knee–ankle angle (HKAA) and weight‐bearing line ratio (WBLR) using full‐length standing radiographs, is the gold standard for evaluating deformities [18, 30]. In HTO, precise preoperative planning that differentiates between femoral, tibial and intra‐articular contributors to varus deformity is essential for achieving optimal correction and ensuring long‐term survivorship [22, 24, 27].

Despite their diagnostic utility, full‐length standing radiographs are not universally accessible in all clinical settings, often requiring specialized equipment and incurring higher costs and greater radiation exposure than do standard knee radiographs [9, 16]. Consequently, the femorotibial angle (FTA), which can be measured using standard short films, remains a common surrogate in daily practice. Establishing a robust correlation between global alignment parameters, such as the HKAA, and local parameters available on standard radiographs could validate the feasibility of estimating the overall alignment without requiring full‐length imaging, thereby streamlining the clinical workflow and reducing the burden on patients and healthcare facilities.

Furthermore, although bony morphology, such as the medial proximal tibial angle (MPTA), lateral distal femoral angle (LDFA) and soft tissue laxity, represented by the joint line convergence angle (JLCA), contribute to varus deformity [8, 10, 29], the dynamic interplay of these factors throughout the disease process remains poorly understood. Therefore, clarifying this stage‐dependent evolution is crucial, as failure to account for soft tissue contributions in late‐stage knee OA may lead to unpredictable surgical outcomes.

This cross‐sectional study aimed to investigate the comprehensive relationships between lower limb alignment parameters, including the HKAA, FTA, WBLR, MPTA, LDFA and JLCA, in an Asian cohort. Specifically, we aimed to assess the predictive value of standard radiographic measures of global alignment and clarify stage‐dependent structural changes in alignment determinants across knee OA severity.

## METHODS

### Participants

This study analysed data from the 2022 cohort of the Iwaki Health Promotion Project, a community‐based initiative dedicated to enhancing life expectancy through preventive medical interventions and health screenings [2, 5, 6, 7, 23]. The research protocol adhered to the ethical principles of the Declaration of Helsinki and received approval from the ethics committee of our institution (reference number: 2025‐042H). The initial study population comprised 737 volunteers (430 women). To assess primary knee OA, we excluded individuals with a history of lower extremity fractures (*n* = 29), rheumatoid arthritis (*n* = 16), knee surgery (*n* = 12) or hip or knee arthroplasty (*n* = 3). Participants with missing radiographic data (*n* = 4) were also excluded. Consequently, 673 participants (273 men) were included in the analysis.

### Radiographic evaluations

Standing digital long‐leg radiographs were obtained from all participants, with both patellae directed forward and the knees fully extended. Radiographs were acquired using a digital radiography system (CXDI‐40EG; Canon Inc.) under the following standardized conditions: cassette‐to‐tube distance, 300 cm; tube voltage, 85 kV; and tube current, 200 mA. During imaging, the participants stood barefoot with their legs closed and patellae facing forward.

Lower limb alignment parameters, including HKAA, FTA, WBLR, MPTA, LDFA and JLCA, were measured radiographically using mediCAD® software version 5.5 (TOYO Corporation) [1, 21]. Radiographic measurements were obtained individually for each knee without accounting for patient demographics or clinical background. Accordingly, measurements for the right and left knees of each participant were treated as independent observations in all statistical analyses, as the primary focus of this study was to evaluate local joint morphology and alignment. HKAA was defined as the angle between the mechanical femoral and tibial axes [15], with positive values indicating varus alignment and negative values representing valgus alignment. FTA was defined as the angle between the femoral and tibial anatomical axes [17]. WBLR was defined as the percentage position of the proximal tibial joint surface along the Mikulicz line (functional axis of the lower limb connecting the centre of the femoral head and the centre of the ankle joint), with values < 50% indicating varus alignment [28]. MPTA was defined as the medial angle between the tibial mechanical axis and the proximal tibial joint line [26]. LDFA was defined as the lateral angle between the femoral mechanical axis and the distal femoral joint line [4]. JLCA was defined as the angle between the distal femoral and proximal tibial joint surfaces [4].

Interobserver agreement was evaluated using intraclass correlation coefficients (ICCs). Two experienced senior orthopaedic surgeons independently measured 50 radiographs (100 knees). ICC (2.1) values were 0.998 (0.997–0.999), 0.992 (0.985–0.995), 0.953 (0.918–0.972), 0.870 (0.775–0.924), 0.921 (0.902–0.964) and 0.876 (0.835–0.913) for HKAA, LDFA, MPTA, JLCA, FTA and WBLR, respectively.

### Knee OA evaluations

OA severity in each knee was classified by two trained orthopaedic surgeons (R. K. and E. S.) using the Kellgren–Lawrence Grade (KLG) 0–4 [14]. The surgeons assessed the KLG without accounting for participants' clinical status.

### Statistical analysis

Descriptive statistics for demographic and radiographic parameters are expressed as means ± standard deviations. The normality of the data distribution was evaluated using the Shapiro–Wilk test. To assess the associations among alignment parameters, Spearman's correlation coefficients were calculated for the entire cohort.

Simple linear regression analyses were performed to determine the quantitative relationships between these parameters. Regression equations were used to distinguish one alignment parameter from another. Furthermore, to clarify the stage‐dependent evolution of deformity determinants, participants were stratified according to the KLG. Correlation analyses and linear regressions were conducted within each subgroup to identify shifts in the contributions of bony morphology and soft tissue laxity to varus alignment. All analyses were performed using SPSS version 29.0 (IBM Corp.). Statistical significance was set at *p* < 0.05.

## RESULTS

The mean participant age, height and body mass index (BMI) were 52.6 ± 15.0 years, 161.6 ± 8.7 cm and 23.0 ± 3.3 kg/m^2^, respectively. KLG distribution was Grades 0, 1, 2, 3 and 4 in 510 (37.9%), 416 (30.9%), 328 (24.4%), 88 (6.5%) and four (0.3%) knees, respectively. For stage‐dependent analyses, knees were categorized as early‐ (KLG 0–1, *n* = 926), progressive‐ (KLG 2, *n* = 328) and end‐stage (KLG 3–4, *n* = 92).

**Figure 1:**
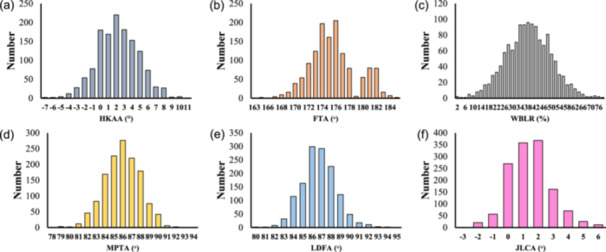
Distribution of coronal alignment parameters in the study population. The bar graph indicates the distribution of the number of participants for each 1° increment in HKAA (a), FTA (b), MPTA (d), LDFA (e) and JLCA (f), and for each 2% increment in WBLR (c). FTA, femorotibial angle; HKAA, hip–knee–ankle angle; JLCA, joint line convergence angle; LDFA, lateral distal femoral angle; MPTA, medial proximal tibial angle; WBLR, weight‐bearing line ratio.

**Figure 2:**
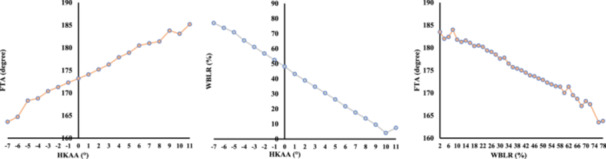
Trends in global alignment parameters relative to deformity progression. The line graphs illustrate the mean values of WBLR and FTA corresponding to each 1° increment in HKAA. FTA, femorotibial angle; HKAA, hip–knee–ankle angle; WBLR, weight‐bearing line ratio.

In the overall cohort, mean HKAA, FTA and WBLR were 2.2° ± 2.6°, 175.6° ± 3.4° and 38.3% ± 11.6%, respectively. Mean MPTA and LDFA were 86.0° ± 2.0° and 86.7° ± 1.9°, respectively. Mean JLCA was 1.5° ± 1.4°. Global alignment parameters indicated significant progression of varus deformity in the end‐stage group (HKAA: 3.5° ± 3.3°; WBLR: 33.3% ± 14.8%) compared with that in the early‐stage group (HKAA: 2.2° ± 2.6°, *p* < 0.001; WBLR: 38.5% ± 11.3%, *p* < 0.001). Among bony parameters, MPTA did not decrease in the end‐stage group (85.8° ± 2.3°) compared with that in the early‐stage group (85.8° ± 2.0°), while LDFA showed no significant change across stages (*p* = 0.35). In contrast, JLCA demonstrated a significant and progressive increase (early: 1.3° ± 1.3° vs. end‐stage: 2.6° ± 1.6°, *p* < 0.001). Figure 1 illustrates the bell‐shaped distribution of HKAA in the study population, with the peak prevalence centreing around neutral to mild varus alignment. Figure 2 demonstrates the synchronized progression of global alignment markers. With an increase in the varus deformity (higher HKAA), there was a linear decrease in WBLR and a linear increase in FTA.

Tables 1, 2, 3 summarize the simple linear regression results and Spearman correlation coefficients for alignment parameters across KLG categories. In the overall cohort, HKAA showed a very strong negative correlation with WBLR (*r* = −0.99, *p* < 0.001) and a strong positive correlation with FTA (*r* = 0.86, *p* < 0.001) (Table 1). In addition to its strong correlation with HKAA, FTA exhibited significant correlations with MPTA (*r* = −0.64, *p* < 0.001), LDFA (*r* = 0.40, *p* < 0.001) and JLCA (*r* = 0.15, *p* < 0.001) (Table 2). Simple linear regression yielded the following predictive equations using FTA: HKAA = 0.65 × FTA − 112.29 and WBLR = −2.86 × FTA + 540.76. In the early‐ and progressive‐stage groups, both MPTA and LDFA showed moderate‐to‐strong correlations with HKAA. In the end‐stage group, JLCA demonstrated a strong correlation with HKAA (*r* = 0.61, *p* < 0.001) (Table 3).

**Table 1:** Regression analyses of global alignment parameters (HKAA, WBLR and FTA).

Overall (*n* = 1346)					KLG 0 and 1 (*n* = 926)			KLG 2 (*n* = 328)			KLG 3 and 4 (*n* = 92)		
*Y*	*X*	*r*	Regression equation	*p* value	*r*	Regression equation	*p* value	*r*	Regression equation	*p* value	*r*	Regression equation	*p* value
HKAA	WBLR	−0.992	−0.224*x* + 10.799	<0.001	−0.992	−0.224*x* + 10.785	<0.001	−0.992	−0.223*x* + 10.721	<0.001	−0.995	−0.225*x* + 10.962	<0.001
WBLR	HKAA	−0.992	−4.392*x* + 48.029	<0.001	−0.992	−4.391*x* + 48.001	<0.001	−0.992	−4.424*x* + 48.040	<0.001	−0.995	−4.391*x* + 48.491	<0.001
HKAA	FTA	0.855	0.652*x* − 112.290	<0.001	0.850	0.649*x* − 111.765	<0.001	0.838	0.633*x* − 108.977	<0.001	0.909	0.681*x* − 116.849	<0.001
FTA	HKAA	0.855	1.121*x* + 173.146	<0.001	0.850	1.113*x* + 173.232	<0.001	0.838	1.109*x* + 173.043	<0.001	0.909	1.214*x* + 172.573	<0.001
WBLR	FTA	−0.847	−2.861*x* + 540.759	<0.001	−0.841	−2.844*x* + 537.953	<0.001	−0.826	−2.783*x* + 526.822	<0.001	−0.915	−3.025*x* + 567.964	<0.001
FTA	WBLR	−0.847	−0.251*x* + 185.240	<0.001	−0.841	−0.249*x* + 185.223	<0.001	−0.826	−0.245*x* + 184.864	<0.001	−0.915	−0.277*x* + 185.985	<0.001

*Note*: The regression equations formulate the predictive relationship where the variable in the ‘*Y*’ column is the dependent variable and that in the ‘*X*’ column is the independent variable. Statistical significance was set at *p* < 0.05.

Abbreviations: FTA, femorotibial angle; HKAA, hip–knee–ankle angle; KLG, Kellgren–Lawrence Grade; WBLR, weight‐bearing line ratio.

**Table 2:** Regression analyses of relationships between global alignment parameters and bony morphology (MPTA and LDFA).

Overall (*n* = 1346)					KLG 0 and 1 (*n* = 926)			KLG 2 (*n* = 328)			KLG 3 and 4 (*n* = 92)		
*Y*	*X*	*r*	Regression equation	*p* value	*r*	Regression equation	*p* value	*r*	Regression equation	*p* value	*r*	Regression equation	*p* value
MPTA	FTA	−0.639	−0.375*x* + 151.842	<0.001	−0.643	−0.383*x* + 152.989	<0.001	−0.591	−0.345*x* + 146.922	<0.001	−0.734	−0.381*x* + 153.159	<0.001
FTA	MPTA	−0.639	−1.087*x* + 269.042	<0.001	−0.643	−1.080*x* + 268.324	<0.001	−0.591	−1.011*x* + 262.724	<0.001	−0.734	−1.414*x* + 298.094	<0.001
HKAA	MPTA	−0.591	−0.768*x* + 68.200	<0.001	−0.617	−0.791*x* + 70.063	<0.001	−0.498	−0.644*x* + 57.731	<0.001	−0.678	−0.979*x* + 87.390	<0.001
MPTA	HKAA	−0.591	−0.456*x* + 86.967	<0.001	−0.617	−0.481*x* + 86.833	<0.001	−0.498	−0.385*x* + 87.248	<0.001	−0.678	−0.470*x* + 87.397	<0.001
MPTA	WBLR	0.580	0.101*x* + 82.089	<0.001	0.603	0.106*x* + 81.703	<0.001	0.480	0.083*x* + 83.220	<0.001	0.698	0.110*x* + 82.124	<0.001
WBLR	MPTA	0.580	3.335*x *− 248.426	<0.001	0.603	3.427*x *− 255.512	<0.001	0.480	2.768*x *− 200.266	<0.001	0.698	4.442*x *− 347.728	<0.001
HKAA	LDFA	0.550	0.775*x *− 64.965	<0.001	0.587	0.836*x *− 70.291	<0.001	0.548	0.683*x *− 57.313	<0.001	0.396	0.656*x *− 53.336	<0.001
LDFA	HKAA	0.550	0.391*x* + 85.853	<0.001	0.587	0.413*x* + 85.790	<0.001	0.548	0.439*x* + 85.956	<0.001	0.396	0.240*x* + 85.813	<0.001
WBLR	LDFA	−0.550	−3.429*x* + 335.630	<0.001	−0.584	−3.681*x* + 357.576	<0.001	−0.562	−3.127*x* + 310.588	<0.001	−0.367	−2.680*x* + 265.513	<0.001
LDFA	WBLR	−0.550	−0.088*x* + 90.099	<0.001	−0.584	−0.093*x* + 90.252	<0.001	−0.562	−0.101*x* + 90.794	<0.001	−0.367	−0.050*x* + 88.316	<0.001
FTA	LDFA	0.398	0.734*x* + 111.998	<0.001	0.432	0.805*x* + 105.882	<0.001	0.382	0.630*x* + 120.582	<0.001	0.271	0.599*x* + 124.912	0.009
LDFA	FTA	0.398	0.215*x* + 48.902	<0.001	0.432	0.232*x* + 46.013	<0.001	0.382	0.231*x* + 46.316	<0.001	0.271	0.123*x* + 64.950	0.009
MPTA	LDFA	0.105	0.114*x* + 76.036	<0.001	0.043	0.048*x* + 81.620	0.187	0.212	0.204*x* + 68.721	<0.001	0.227	0.260*x* + 63.210	0.0295
LDFA	MPTA	0.105	0.097*x* + 78.365	<0.001	0.043	0.039*x* + 83.329	0.187	0.212	0.220*x* + 67.841	<0.001	0.227	0.198*x* + 69.660	0.0295

*Note*: The regression equations formulate the predictive relationship where the variable in the ‘*Y*’ column is the dependent variable and the variable in the ‘*X*’ column is the independent variable. Statistical significance was set at *p* < 0.05.

Abbreviations: FTA, femorotibial angle; HKAA, hip–knee–ankle angle; KLG, Kellgren–Lawrence Grade; LDFA, lateral distal femoral angle; MPTA, medial proximal tibial angle; WBLR, weight‐bearing line ratio.

**Table 3:** Regression analyses of relationships between global alignment parameters and soft tissue laxity (JLCA).

Overall (*n* = 1346)					KLG 0 and 1 (*n* = 926)			KLG 2 (*n* = 328)			KLG 3 and 4 (*n* = 92)		
*Y*	*X*	*r*	Regression equation	*p* value	*r*	Regression equation	*p* value	*r*	Regression equation	*p* value	*r*	Regression equation	*p* value
WBLR	JLCA	−0.287	−2.367*x* + 41.703	<0.001	−0.213	−1.831*x* + 40.816	<0.001	−0.300	−2.339*x* + 42.887	<0.001	−0.603	−5.452*x* + 47.401	<0.001
JLCA	WBLR	−0.287	−0.035*x* + 2.785	<0.001	−0.213	−0.025*x* + 2.220	<0.001	−0.300	−0.039*x* + 3.150	<0.001	−0.603	−0.067*x* + 4.810	<0.001
HKAA	JLCA	0.286	0.532*x* + 1.450	<0.001	0.203	0.395*x* + 1.664	<0.001	0.309	0.541*x* + 1.145	<0.001	0.605	1.240*x* + 0.253	<0.001
JLCA	HKAA	0.286	0.153*x* + 1.111	<0.001	0.203	0.105*x* + 1.043	<0.001	0.309	0.177*x* + 1.285	<0.001	0.605	0.295*x* + 1.568	<0.001
FTA	JLCA	0.151	0.368*x* + 175.103	<0.001	0.086	0.219*x* + 175.364	<0.001	0.133	0.308*x* + 174.791	0.016	0.495	1.355*x* + 173.267	<0.001
JLCA	FTA	0.151	0.062*x *− 9.385	<0.001	0.086	0.034*x *− 4.660	<0.001	0.133	0.058*x *− 8.468	0.016	0.495	0.181*x *− 29.423	<0.001

*Note*: The regression equations formulate the predictive relationship where the variable in the ‘*Y*’ column is the dependent variable and the variable in the ‘*X*’ column is the independent variable. Statistical significance was set at *p* < 0.05.

Abbreviations: FTA, femorotibial angle; HKAA, hip–knee–ankle angle; JLCA, joint line convergence angle; KLG, Kellgren–Lawrence Grade; WBLR, weight‐bearing line ratio.

**Figure 3:**
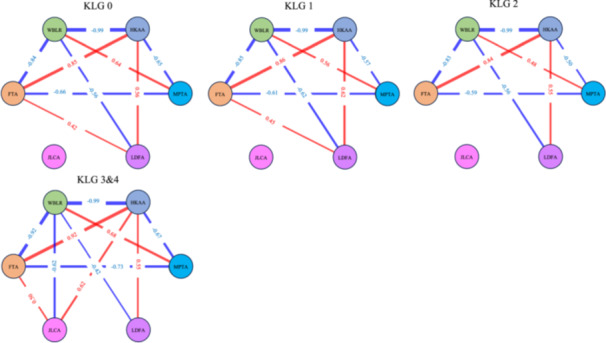
Correlation networks of alignment parameters across KLGs. Network diagrams visualizing the structural relationships among lower limb alignment parameters for each KLG subgroup. Nodes represent alignment parameters (HKAA, WBLR, FTA, MPTA, LDFA, JLCA), and edges (lines) represent Spearman's correlation coefficients with an absolute value greater than 0.40 (*r* > 0.40). FTA, femorotibial angle; HKAA, hip–knee–ankle angle; JLCA, joint line convergence angle; KLG, Kellgren–Lawrence Grade; LDFA, lateral distal femoral angle; MPTA, medial proximal tibial angle; WBLR, weight‐bearing line ratio.

Figure 3 displays the correlation networks for each KLG subgroup. In the early‐ and progressive‐stage networks, JLCA nodes were relatively isolated, indicating a limited contribution to the overall alignment network. In contrast, the end‐stage networks showed thick, strong edges connecting JLCA with HKAA and WBLR.

## DISCUSSION

The primary finding of this study quantitatively confirmed the stage‐dependent shift in the structural determinants of varus alignment. Our data provide robust quantitative evidence reinforcing the established concept that the pathology progresses from a ‘bony‐driven’ condition to a ‘combined bony‐ and soft tissue‐driven’ condition.

In the general context of lower limb alignment, our results reaffirmed a robust linear relationship between HKAA and WBLR (*r* = −0.99). Regression analysis indicated that a 1° change in HKAA corresponded to an approximately 4.4% shift in WBLR. This finding aligns with previous reports, which generally suggest that a 1° varus angulation shifts WBLR medially by approximately 3%–5% in HTO surgeries [11, 13]. Furthermore, we formulated predictive equations using FTA, a parameter that is readily measurable on standard anteroposterior (AP) knee radiographs. As visualized in Figure 2, the trajectory of FTA closely mirrors that of HKAA and WBLR across all deformity strata. This strong linear alignment, supported by the correlation coefficient of 0.86, robustly supports the use of FTA as a reliable surrogate for estimating global alignment. The graphical evidence in Figure 2 reinforces that even in standard clinical settings without full‐length imaging, FTA provides a consistent reflection of the mechanical loading environment. This validation is clinically useful for initial screening in primary care settings, where access to specialized imaging equipment may be limited. However, a specific characteristic of FTA in end‐stage disease must be noted. While HKAA demonstrated a strong correlation with soft tissue laxity (JLCA) in the end‐stage group (*r* = 0.62), the correlation between FTA and JLCA was moderate (*r* = 0.50). This discrepancy arises from geometric definitions; HKAA represents the mechanical axis, which directly incorporates the joint line opening (JLCA) as a linear deviation of the weight‐bearing line. In contrast, FTA represents the anatomical angle between the femoral and tibial shafts. Therefore, while FTA effectively tracks the resultant deformity, it is theoretically less sensitive to the pure soft tissue component than is HKAA. Clinicians should be aware that compared with full‐length imaging, FTA may slightly underestimate the magnitude of the soft tissue contribution in knees with severe varus deformity.

JLCA is widely recognized as a radiographic surrogate that reflects not only lateral soft tissue laxity and joint instability, but also cartilage degeneration and meniscal extrusion [4, 19]. Tsushima et al. reported that JLCA reflected KLG, meniscal status and cartilage damage [29]. In our correlation analysis, JLCA was isolated in the early stages, suggesting that joint laxity was not the primary driver of deformity at disease onset. Conversely, in the end‐stage group, JLCA formed strong connections with HKAA and WBLR, consistent with pathological progression in which cartilage loss and lateral laxity secondary to chronic varus stress lead to joint instability. Previous studies have primarily focused on bony deformities (i.e., progression of varus deformity in the proximal tibia) [8]; nonetheless, our data indicate that, in end‐stage OA, intra‐articular conditions become as critical as bony components. These findings have important implications for preoperative planning of joint‐preserving surgeries, particularly HTO. In patients with end‐stage OA who are candidates for osteotomy, relying solely on bony correction based on MPTA may yield inaccurate alignment targets [12]. Because JLCA represents a dynamic, often correctable, soft tissue component, ignoring its contribution could result in overcorrection if the surgical plan addresses the total varus deformity purely through bone osteotomy [20]. Therefore, surgeons should carefully assess the contribution of JLCA, particularly in severe cases.

Regarding the study cohort, we applied strict exclusion criteria such as a history of lower limb fractures or rheumatoid arthritis. These conditions can fundamentally alter joint mechanics and bone morphology independent of the degenerative process. By excluding them, we minimized confounding bias, ensuring that our analysis reflected the natural morphological changes associated with primary OA progression.

This study had some limitations. First, the cross‐sectional design precluded causal inference regarding disease progression; longitudinal studies are needed to determine whether increased JLCA precedes or follows progression to end‐stage OA. Second, radiographic measurements were performed on two‐dimensional (2D) images, which do not account for rotational deformities or flexion contractures that may influence alignment assessment. Third, the study population comprised a specific Japanese cohort, potentially limiting generalizability to other ethnic groups with different anthropometric characteristics. Specifically, geometric considerations indicate that the relationship between FTA and HKAA is influenced by lower‐extremity length. Fourth, our study design did not strictly differentiate between constitutional varus alignment and pathological varus deformity, particularly in the early OA stages. Because this was a cross‐sectional analysis, we could not determine whether the varus alignment observed in participants with low KLG was a stable constitutional phenotype or a precursor to progressive OA. Consequently, the relationships among alignment parameters observed in the early stages may reflect the characteristics of the varus phenotype rather than the pathological process of OA progression. Longitudinal data are required for the transition from constitutional alignment to pathological deformity. Finally, the number of participants in the end‐stage group was relatively small compared with that in the early‐stage group. This distribution reflected the natural prevalence of advanced OA in a general community population, which differs from that in hospital‐based cohorts. While this sample size limited the statistical power for subgroup analyses of patients with severe disease, we prioritized use of a pure community‐based sample to avoid the selection bias associated with clinical populations.

In conclusion, the determinants of varus deformity in knee OA are not static but evolve with disease stage. While bony morphology is the primary driver in the early stages, the JLCA is a key end‐stage OA determinant. Understanding this structural shift is essential for accurate clinical assessment and surgical planning. Our findings validate the utility of standard radiographs for estimating global alignment, bridging the gap between specialized imaging and daily clinical practice.

## AUTHOR CONTRIBUTIONS

Kyota Ishibashi participated in the study design, supervised data collection and drafted the manuscript. Ryoto Kura, Hikaru Kristi Ishibashi, Eiji Sasaki, and Yuka Kimura assisted with statistical analysis and manuscript drafting and critically revised the manuscript. Ryo Tomita, Eiichi Tsuda, and Yasuyuki Ishibashi conceived and designed the study. All authors have read and approved the final manuscript.

## CONFLICT OF INTEREST STATEMENT

The authors declare no conflict of interest.

## ETHICS STATEMENT

This study was conducted in accordance with the Declaration of Helsinki (1964) and its later amendments and was approved by the ethics committee of our institution (reference number: 2025‐042H). Written informed consent was obtained from all participants before enrolment.

## Data Availability

Data are available on request from the authors.
